# Cortical speech tracking is related to individual prediction tendencies

**DOI:** 10.1093/cercor/bhac528

**Published:** 2023-01-09

**Authors:** Juliane Schubert, Fabian Schmidt, Quirin Gehmacher, Annika Bresgen, Nathan Weisz

**Affiliations:** Centre for Cognitive Neuroscience and Department of Psychology, University of Salzburg, Austria; Centre for Cognitive Neuroscience and Department of Psychology, University of Salzburg, Austria; Centre for Cognitive Neuroscience and Department of Psychology, University of Salzburg, Austria; Centre for Cognitive Neuroscience and Department of Psychology, University of Salzburg, Austria; Centre for Cognitive Neuroscience and Department of Psychology, University of Salzburg, Austria; Neuroscience Institute, Christian Doppler University Hospital, Paracelsus Medical University, Salzburg, Austria

**Keywords:** auditory processing, cortical speech tracking, interindividual differences, magnetoencephalography, predictive processing

## Abstract

Listening can be conceptualized as a process of active inference, in which the brain forms internal models to integrate auditory information in a complex interaction of bottom-up and top-down processes. We propose that individuals vary in their “prediction tendency” and that this variation contributes to experiential differences in everyday listening situations and shapes the cortical processing of acoustic input such as speech. Here, we presented tone sequences of varying entropy level, to independently quantify auditory prediction tendency (as the tendency to anticipate low-level acoustic features) for each individual. This measure was then used to predict cortical speech tracking in a multi speaker listening task, where participants listened to audiobooks narrated by a target speaker in isolation or interfered by 1 or 2 distractors. Furthermore, semantic violations were introduced into the story, to also examine effects of word surprisal during speech processing. Our results show that cortical speech tracking is related to prediction tendency. In addition, we find interactions between prediction tendency and background noise as well as word surprisal in disparate brain regions. Our findings suggest that individual prediction tendencies are generalizable across different listening situations and may serve as a valuable element to explain interindividual differences in natural listening situations.

## Introduction

Listening is a neurobiological challenge that requires a complex interaction of bottom-up and top-down processes. For instance, understanding speech in a noisy environment by bottom-up input alone would be impossible because of the vast amount of overlapping spectrotemporal information (McDermott 2009). In line with notions of the so-called predictive brain (Knill and Pouget 2004; Friston 2010; Yon et al. 2019; Friston et al. 2021), we assume that our brain is actively engaged when listening to speech by fitting and testing experience-based internal models, inferring which sound sources (“auditory objects”; Griffiths and Warren 2004) are causing the neural activity patterns. This process requires the constant generation of predictions that are continuously compared with incoming bottom-up information. We argue that interindividual differences in the tendency to use predictions exist, and that these differences have consequences for real-life listening experience such as speech perception.

Indeed, “normal” hearing individuals vary considerably in their ability to understand speech with some even reporting difficulties in everyday speech comprehension and communication (Ruggles et al. 2011). In search of a predictor of linguistic abilities, individual differences in statistical learning, “a general capacity for picking up regularities,” have already been proposed (for a review see Siegelman et al. 2017). Crucially, however, statistical learning as an individual capacity should be operationalized and precisely which of its components drive the relationship between statistical learning and linguistic performance remains unclear. We suggest that the individual tendency to actively generate auditory predictions might be one such factor. We propose that this tendency can even be conceptualized as a “trait-like” disposition, following a notion that has already been used to explain clinical psychological conditions and disorders such as autism (Sinha et al. 2014), schizophrenia (Sterzer et al. 2018), and tinnitus (Sedley et al. 2016; Partyka et al. 2019). Although strong predictive tendencies can promote illusory auditory perceptions (Powers et al. 2017), overall they should be adaptive in listening situations as it has been shown that speech-auditory cortex coupling is enhanced as a function of stronger top-down signals (Park et al. 2015). Here we aim to establish empirically how interindividual variability in “prediction tendency,” which we operationalize as the tendency to preactivate sensory features of strong likelihood, contributes to differences in speech processing.

Another goal of the current study is to detail the circumstances under which strong prediction tendencies are particularly beneficial for speech processing. Interindividual differences become especially apparent in noisy situations, which are typical for natural environments (Meyer et al. 2013). In the iconic cocktail party situation multiple speech streams enter the auditory system, yet only one is to be followed (Oberfeld and Klöckner-Nowotny 2016). This task gets increasingly challenging with increased interfering noise, revealing and/or increasing interindividual differences in listening experiences (Wöstmann et al. 2015). Regardless of signal-to-noise ratio, it should be considered that even under optimal conditions not all incoming sensory information is equally predictable. Thus, how the relationship between prediction tendency and speech processing depends on the predictability of the speech input itself is another important question that needs to be addressed. Although there are several neuroimaging studies offering valuable insight into the effect of prior knowledge on isolated sentences (Di Liberto et al. 2018) or target words (Sohoglu et al. 2012), it has been questioned whether effects from controlled manipulation generalize to natural, narrative language. More recent studies, that investigated speech processing on a continuous scale, also suggest an influential role of speech predictability in general (e.g. semantic context and word surprisal (Broderick et al. 2019; Donhauser and Baillet 2020; Weissbart et al. 2020).

**Fig. 1:**
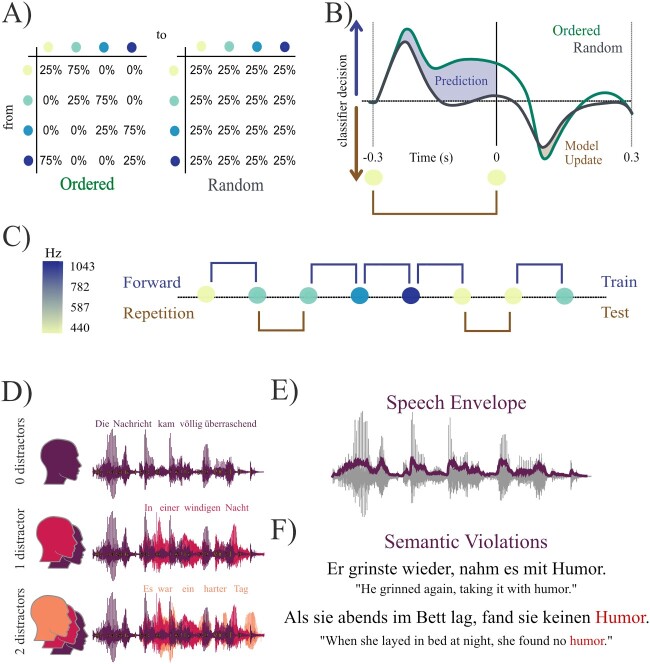
A–C): Quantification of individual prediction tendency. A) Participants have been presented with sequences of 4 different pure tones at a rate of 3 Hz. Transitional probabilities varied according to 2 different entropy conditions (ordered vs. random). C) Example of an ordered sound sequence. An LDA classifier was used to decode sound frequency from brain activity across time, trained on ordered forward transition trials and tested on all repetition trials. B) Expected classifier decision values contrasting the brains pre-stimulus tendency to predict a forward transition and post-stimulus correction for the actually presented repetition. Individual prediction and model updating quantification result from the difference between conditions (ordered > random). D–F): Multi speaker paradigm. D) Subjects were instructed to listen to a “target speaker” (purple). Depending on the experimental condition, the target speech stream was disrupted by 1 or 2 additional distracting speakers. E) The speech envelope of the target speaker was extracted and used to calculate a TRF (Brodbeck et al. 2021) with the associated brain activity recorded using MEG. F) Semantic violations were introduced randomly by replacing the last noun of a sentence with an improbable candidate to measure the effect of envelope encoding in conjunction with prediction tendency on the processing of semantic violations.

In the current study, we combined magnetoencephalographic (MEG) data from 2 different paradigms in order to establish a link between individual prediction tendency and speech perception: (i) An adapted paradigm as used by Demarchi et al. (2019), presenting tone sequences of varying entropy level (in the following referred to as entropy modulation paradigm), which was used to independently quantify individual prediction tendency (for details see Fig. 1A–C). (ii) A multi speaker listening task to investigate individual differences in cortical speech tracking (i.e. tracking of the acoustic envelope, that is extracted from the audiofile and used as a predictor to explain variance in the neural signal) across different levels of background noise. We used natural, continuous speech but also introduced rare semantic violations by randomly replacing a limited set of words with target words that occurred elsewhere in the same storyline (see Fig. 1D–F). This allowed us to investigate encoding of the acoustic envelope on a continuous scale as well as on an individual word level (of lexically identical stimuli), ensuring that effects of word surprisal cannot be attributed to differences in bottom-up input. Our results show that cortical speech tracking in perisylvian and inferior frontal areas of both hemispheres is enhanced in individuals with strong prediction tendency, which we define as the tendency to anticipate auditory events of high probability. Furthermore, we find spatially dissociable interactions between individual prediction tendency and background noise as well as word surprisal. In sum, these findings suggest that individual prediction tendencies show considerable generalizability across different listening situations and may serve as a valuable element to explain interindividual differences in natural listening experience.

## Methods

### Subjects

In total 53 (21 male) subjects were recruited to participate in the study, however 4 subjects did not complete the experiment due to technical difficulties, leaving a total number of 49 subjects (mean age = 26.31, range = 19–40) for further data analysis. Originally we aimed for a total of 60 participants to enable a meaningful between-subject analysis, however due to unexpected coronavirus disease 2019 (COVID-19) related restrictions and limited access to our laboratories we decided to stop measurements after 2 years before reaching the anticipated sample size. Participation was compensated either monetarily or via course credits. All participants were German native speakers and reported normal hearing, which was confirmed using standard pure tone audiometry. Participants gave written, informed consent and reported that they had no previous neurological or psychiatric disorders. The experimental procedure was approved by the ethics committee of the University of Salzburg and was carried out in accordance with the declaration of Helsinki.

### Stimuli

We used audio recordings of excerpts from German books and short-stories (Supplementary Table S1, see online supplementary material). In total, 6 different, consistent target stories (à ~ 10 min) and 18 distractor stories (à ~3 min) were recorded with a t.bone SC 400 studio microphone and a sampling rate of 44,100 Hz. Prior to recording, target stories were split into separate trials of ~3–4 min (mean = 3.46 min, range = 3.05–4.07 min). In addition, we randomly selected half of the nouns that ended a sentence and replaced them with the other half to induce unexpected semantic violations within each trial, resulting in 2 sets of lexically identical words (*N* = 79) that differed greatly in their contextual probabilities (see Fig. 1F for an example). All 6 target stories were recorded twice, narrated by a different speaker (male vs. female). The remaining 18 recordings were narrated by the same 2 speakers (used as second female/male distractor speaker to a male/female target speaker, respectively) and 2 additional speakers (used as first distractor speaker). Stimuli were presented in 6 blocks containing 3 trials each, resulting in 3 male and 3 female target speaker blocks for every participant (see section “Experimental procedure”).

### Experimental procedure

Before the start of the experiment, we performed standard pure tone audiometry using the AS608 Basic (Interacoustics, Middelfart, Denmark) to assess participants’ individual hearing ability. Afterwards, participants’ individual head shapes were assessed using cardinal head points (nasion and preauricular points), digitized with a Polhemus Fastrak Digitiser (Polhemus) at around 300 points on the scalp. For every participant MEG sessions started with a 5-min resting state recording, after which the individual hearing threshold was determined using a pure tone of 1,043 Hz. This was followed by 2 blocks of passive listening to tone sequences of varying entropy level to quantify individual prediction tendencies (see quantification of individual prediction tendency) while participants watched a landscape movie. In the main task, 6 different stories were presented in separate blocks in random order and with randomly balanced selection of the target speaker (male vs. female voice). Each block consisted of 3 trials with a continuous storyline, with each trial corresponding to 1 of 3 experimental conditions: a single speaker only, a 1-distractor speaker, and a 2-distractor speaker condition (in the following: 0-dist, 1-dist, and 2-dist, see Fig. 1D). The distractor speakers were selected to be of the opposite sex of the target speaker and were presented exactly 20 s after target speaker onset. All stimuli were presented binaurally at equal volume for the left and right ear (i.e. at phantom center). Participants were instructed to attend to the first speaker and their understanding was tested using comprehension questions (true vs. false statements) at the end of each trial. Furthermore, participants indicated their task engagement and their perceived task-difficulty on a 5-point Likert scale at the end of every trial. All stimuli were presented at 40-db above the individual hearing threshold. In total, the experiment lasted ~3.5 h per participant (including MEG preparation time). The experiment was coded and conducted with the Psychtoolbox-3 (Brainard 1997; Kleiner et al. 2007) with an additional class-based library (“Objective Psychophysics Toolbox,” o_ptb) on top of it (Hartmann and Weisz 2020).

### MEG data acquisition and analysis

A whole head MEG system (Elekta Neuromag Triux, Elekta Oy, Finland), placed within a standard passive magnetically shielded room (AK3b, Vacuumschmelze, Germany), was used to capture magnetic brain activity with a sampling frequency of 10 kHz (hardware filters: 0.1–3,300 Hz). The signal was recorded with 102 magnetometers and 204 orthogonally placed planar gradiometers at 102 different positions. In a first step, a signal space separation algorithm, implemented in the Maxfilter program (version 2.2.15) provided by the MEG manufacturer, was used to clean the data from external noise and realign data from different blocks to a common standard head position. Data preprocessing was performed using Matlab R2020b (The MathWorks, Natick, MA, United States) and the FieldTrip Toolbox (Oostenveld et al. 2010). All data were filtered between 0.1 and 30 Hz (Kaiser windowed finite impulse response filter) and downsampled to 100 Hz. To identify eye-blinks and heart rate artifacts, 50 independent components were identified from filtered (0.1–100 Hz), downsampled (1,000 Hz) continuous data of the recordings from the entropy modulation paradigm and on average 3 components were removed for every subject. Data of the entropy modulation paradigm were epoched into segments of 1,200 ms (from 400 ms before sound onset to 800 ms after onset). Multivariate pattern analysis (see section ``Quantification of individual prediction tendency'') was carried out using the MVPA-Light package (Treder 2020). Single trial data from the main task were further projected into source-space using LCMV spatial filters (Van Veen et al. 1997). To compute these filters, anatomical template images were warped to the individual head shape and brought into a common space by co-registering them based on the 3 anatomical landmarks (nasion, left, and right preauricular points) with a standard brain from the Montreal Neurological Institute (MNI, Montreal, Canada; Mattout et al. 2007). Afterwards, a single-shell head model (Nolte 2003) was computed for each participant. As a source model, a grid with 1-cm resolution and 2,982 voxels based on an MNI template brain was morphed into the brain volume of each participant. This allows group-level averaging and statistical analysis as all the grid points in the warped grid belong to the same brain region across subjects. Afterwards, the data were temporally aligned with the corresponding features from the audio material.

### Quantification of individual prediction tendency

In the present study, we used an entropy modulation paradigm where participants passively listened to sequences of 4 different pure tones (f1: 440 Hz, f2: 587 Hz, f3: 782 Hz, and f4: 1,043 Hz, each lasting 100 ms) during 2 separate blocks, each consisting of 1,500 tones presented with a temporally predictable rate of 3 Hz (adapted from Demarchi et al. 2019). Entropy levels (ordered/random) changed between the 2 blocks for half of the participants and pseudorandomly every 500 trials within each block for the other half, always resulting in a total of 1,500 trials per entropy condition. By design, the transitional probabilities between tones varied according to the different levels of entropy (ordered vs. random; see Fig. 1A): While in an “ordered” context certain transitions (hereinafter referred to as forward transitions, i.e. f1 → f2, f2 → f3, f3 → f4, and f4 → f1) were to be expected with a high probability of 75%, self-repetitions (e.g. f1 → f1 and f2 → f2,...) were rather unlikely with a probability of 25%. However, in a “random” context all possible transitions (including forward transitions and self-repetitions) were equally likely with a probability of 25%. As Demarchi et al. (2019) showed that preactivation of carrier-frequency related neural activity is systematically enhanced within low entropy conditions, it can be assumed that correct frequency-specific predictions about upcoming events should only be generated for transitions of high probability, i.e. (expected) forward transitions in an ordered context. Within the same ordered context, the less expected self-repetition trials should erroneously generate pre-stimulus predictions about an upcoming tone in the expected forward direction. As the tone instead is repeated, this expectation is violated which should then lead to post-stimulus model updating due to the unanticipated event. To test this assumption, we used a multiclass linear discriminant analyzer (LDA) to decode sound frequency from brain activity in a time-resolved manner. To capture any prediction related neural activity, the classifier was trained only on forward transition trials of the ordered condition (and thus “predictable” trials) and then tested on self-repetition trials of both entropy conditions. This resulted in classifier decision values (dvals) for every possible sound frequency (d1, d2, d3, and d4) of all test trials (*t*) which were then transformed into corresponding transitions with respect to the preceding sound (*t* − 1) (e.g. d1(*t*) | f1(*t* − 1) “dval for f1 at trial *t* given that f1 was presented at trial *t* − 1” → repetition, d2(*t*) | f1(*t* − 1) → forward,...). This was done for test trials of the ordered and the random entropy condition separately (see Fig. 1C). Resulting classifier decisions for a forward transition vs. a repetition in each repetition trial were contrasted for every time-point from −0.3 to 0.3 s. Then, the average forward-vs.-repetition tendency over trials was zero-centered (resulting in positive values for a forward and negative values for repetition decision) and compared between entropy conditions (tested on ordered vs. tested on random repetitions; see Fig. 1B). Since the classifier is trained on forward transitions only, bottom-up representations of the preceding sound, which fall within the pre-stimulus interval of interest, also result in strongest dvals for a forward transition (i.e. “carry-over classification effect,” which is visible for both conditions as in Fig. 1B). Since we have the same transitions (self-repetitions) in both conditions, we ensure an equal carry-over effect across conditions, which enables us to isolate pre-stimulus prediction effects by contrasting “ordered” vs. “random.” Thus, we quantified “prediction tendency” as the classifiers pre-stimulus decision in favor of a forward transition in an ordered compared with a random context (see Fig. 1B). Analogously, we further quantified “updating tendency” as the post-stimulus decision tendency towards the actually presented repetition transition (again for ordered > random). Using the summed difference across time (pre-stimulus for prediction and post-stimulus for model updating) one value can be extracted per individual subject. Note that condition contrasting was also applied to ensure that individual differences in prediction tendency cannot be explained by subject specific signal-to-noise ratio (as no vacuous difference in SNR is to be expected between conditions). In addition, we used frequency decoding accuracy as a control variable to correlate with speech tracking variables of interest (see Supplementary material).

### Encoding of acoustic features

Firstly, the speech envelope was extracted from the audio files of the target speaker using the Chimera toolbox (Smith et al. 2002) over a broadband frequency range of 100 Hz–10 kHz (in 9 steps, equidistant on the tonotopic map of auditory cortex, see Fig. 1E). Afterwards, to quantify the neural representations corresponding to the acoustic envelope, we calculated a multivariate temporal response function (TRF) using the Eelbrain toolkit (Brodbeck et al. 2021). A deconvolution algorithm (boosting; David et al. 2007) was applied to the concatenated trials of the clear speech condition (0-dist) to estimate the optimal TRF to predict the brain response from the speech envelope for each individual virtual channel in source-space (2,982 voxels). The defined time-lags to train the model were from −100 to 500 ms. To evaluate the model, the 0-dist data was split into 4 folds, and a cross-validation approach was used to avoid overfitting (Ying 2019). Furthermore, the same model was used to deconvolve speech envelope and brain data of the other 2 conditions (1-dist and 2-dist). The resulting predicted channel responses for all conditions were then correlated with the true channel responses in order to quantify the model fit and the degree of speech envelope tracking in a particular brain region.

To investigate the effect of semantic violations the whole data was segmented into single word epochs of 2 s starting at word onset (using a forced-aligner; Kisler et al. 2017; Schiel and Ohala 1999) and grouped into 3 different categories: high surprisal, low surprisal controls (see section Stimuli or Fig. 1F for an example), and all remaining. Words were then concatenated within each category (and distractor condition) before deconvolution was applied and a speech envelope encoding model was estimated from the remaining words category in the clear speech condition (0-dist). Using the same time-lags (−100 to 500 ms) as in the standard analysis, the optimal TRFs were then used to test speech envelope encoding for high surprisal words and (lexically identical low surprisal) controls respectively (again for each individual virtual channel in source-space).

### Statistical analysis

To investigate the influence of individual prediction tendencies on speech tracking under different noise conditions, we used Bayesian multilevel regression models with Bambi (Capretto et al. 2022), a python package built on top of the PyMC3 package (Salvatier et al. 2016) for probabilistic programming. The correlation between predicted brain activity from speech envelope encoding and true brain activity was used as dependent variable, and separate models were calculated per voxel using the following formula according to the Wilkinson notation (Wilkinson and Rogers 1973):

cortical tracking ~ n_distractors ^*^ prediction_tendency + (1|subject_id).

To investigate the influence of higher-level probabilistic structure of speech, we also calculated a model for which the dependent variable only included cortical tracking (i.e. speech envelope encoding) results of lexically identical nouns of high vs. low surprisal:

cortical tracking ~ n_distractors ^*^ word-surprisal ^*^ prediction_tendency  + (1|subject_id).

Before entering the models, prediction_tendency was zero-centered (note that in these interaction models other predictors are assumed to be zero to estimate effects of one predictor) and the number of distractors (0–2) was treated as a continuous variable. As priors we used the weakly- or non-informative default priors of Bambi (Capretto et al. 2022) and specified a more robust Student-*T* distribution as response distributions instead of the default gaussian distribution. For a summary of model parameters we report regression coefficients and 94% high density intervals (HDI; the default HDI in bambi) of the posterior distribution. From the HDIs we can conclude that there is a 94% probability that the respective parameter falls within this interval given the evidence provided by the data, prior and model assumptions. Effects were considered significantly different from zero if the HDI did not include zero. Furthermore, it was ensured that for all models there were no divergent transitions ($\hat{\rm r} $ < 1.05 for all relevant parameters) and an effective sample size > 400 (an exhaustive summary of bayesian model diagnostics can be found in Vehtari et al. 2021). Since separate models were calculated per voxel, to further reduce the possibility of spatially spurious results, voxels were only considered as significant if a minimum of 2 significant neighbors could be detected. Subsequently, posterior distributions were aggregated over voxels that formed a valid cluster (in that sense) to report and plot summary statistics for each effect. Labels for all voxels were obtained using a template atlas (Tzourio-Mazoyer et al. 2002).

Due to an error in the experimental code that resulted in loss of data on accuracy for true vs. false statements, we were not able to investigate listening comprehension on a behavioral level. To investigate the influence of background noise and individual prediction tendency on subjective perception we calculated 2 bayesian multilevel models using the following formulas:

difficulty ~ n_distractors ^*^ prediction_tendency + (1|subject_id).

engagement ~ n_distractors ^*^ prediction_tendency + (1|subject_id).

As dependent variables we used perceived task-difficulty as well as self-reported engagement (mean subjective ratings over blocks on a 5-point likert scale).

## Results

### Quantifying individual prediction tendencies

Firstly, we used an entropy modulation paradigm to quantify the individual prediction tendency, which we define as the tendency to preactivate sound frequencies of high probability (i.e. a forward transition from one tone to another: i.e. f1 → f2, f2 → f3, f3 → f4, and f4 → f1). To estimate this tendency we trained a classifier (LDA) to decode sound frequencies from brain activity in ordered forward trials, in order to capture any prediction related neural activity. Afterwards, the classifier was tested on all self-repetition trials, providing classifier decision values for every sound frequency, which were then transformed into corresponding transitions (e.g. d1(*t*) | f1(*t* − 1) “dval for *f1* at trial *t* given that *f1* was presented at trial *t* − 1” → repetition, d2(*t*) | f1(*t* − 1) → forward, ...). Within an ordered context the unexpected self-repetition trials should erroneously generate pre-stimulus predictions for an upcoming tone in the expected forward direction (in a similar fashion as in ordered forward trials), which then lead to post-stimulus model updating due to the unanticipated repetition event. The tendency to represent a forward-vs.-repetition transition was contrasted for both ordered and random trials. Using self-repetition trials for testing, we ensured a fair comparison between the ordered and the random context (with an equal number of trials as well as the same preceding bottom-up input). Thus, we quantified “prediction tendency” as the classifier’s pre-stimulus tendency to a forward transition in an ordered context exceeding the same tendency in a random context (which can be attributed to carry-over processing of the preceding sound).

**Fig. 2:**
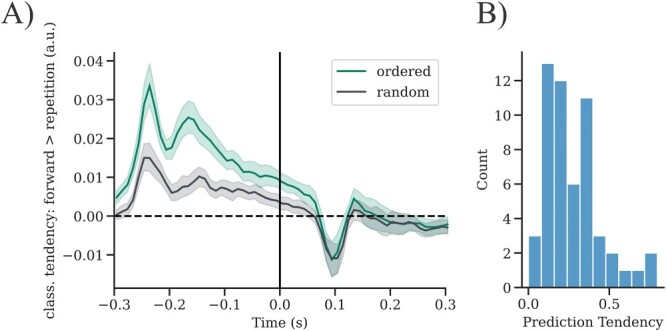
Individual prediction tendency. A) Time-resolved contrasted classifier decision: forward > repetition for ordered and random repetition trials. Classifier tendencies showing frequency-specific prediction for tones with the highest probability (forward transitions) can be found even before stimulus onset but only in an ordered context (shaded areas always indicate 95% confidence intervals). Using the summed difference across pre-stimulus time, one prediction value was extracted per individual subject. B) Distribution of prediction tendency values across subjects (*N* = 49).

Results for quantification of individual prediction tendencies are shown in Fig. 2. We find a clear pre-stimulus tendency to predict a forward transition during repetition trials in the ordered condition, which exceeds the carry-over effect (see Fig. 2). However, the data shows no visible difference in the post-stimulus correction for the actually presented repetition between an ordered and a random context. It is possible that our classifier was unable to identify or use any activity related to prediction violations, as it was trained only on trials, for which the outcome was as predicted (i.e. ordered forward trials). A different approach (e.g. to train on ordered repetition trials) might be more appropriate to capture post-stimulus differences in model updating tendencies. Model updating quantification was therefore dropped and only prediction tendency was used for further analysis.

### Speech envelope encoding is modulated by individual prediction tendency and background noise

**Fig. 3:**
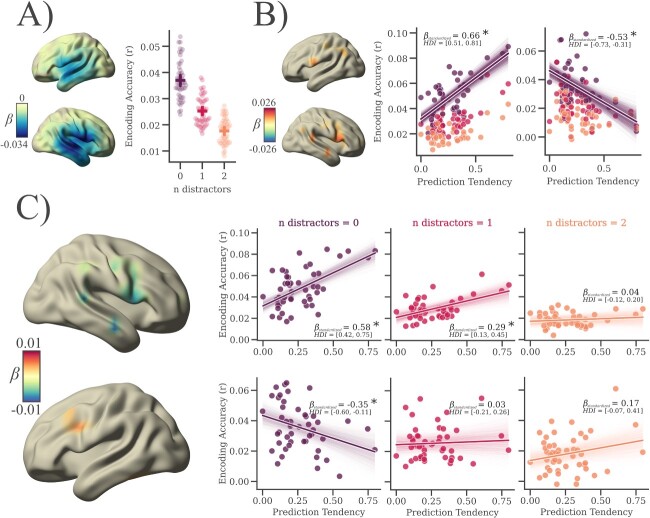
Speech envelope encoding is modulated by individual prediction tendency and background noise. A) The number of distracting speakers negatively affects speech envelope encoding, as background noise increases the encoding accuracy of the speech envelope decreases. B) Individual prediction tendency is positively associated with encoding of clear, continuous speech in bilateral perisylvian areas. There was also a negative effect in the left ITC (the effect, however, being too small to be visible in the brain plot; the purple line indicates the main effect—with other predictors set to zero). C) As background noise increases, effects of prediction tendencies on speech envelope encoding are decreasing (brain maps show “clusters” significantly different from zero, scatter plots show one-directional model results averaged over all clusters—for details see Methods; shaded areas always indicate 94% HDIs, *N* = 49).

In order to investigate the relationship between individual prediction tendencies and speech tracking, we used a multi speaker listening task, where 6 different stories were presented in separate blocks. Each block consisted of 3 trials with a continuous storyline, with each trial corresponding to 1 of 3 experimental conditions: a single speaker only, a 1-distractor speaker, and a 2-distractor speaker condition (in the following: 0-dist, 1-dist, and 2-dist, see Fig. 3A). The distractor speakers were always selected to be of the opposite sex of the target speaker. We find an effect for the number of distractors on self-reported difficulty, indicating that the task is perceived more difficult as the number of distracting speakers increases. This result confirms that our modulation of the background noise achieves the desired effect. A more detailed description of all subjective task ratings (along with figures and summary statistics) can be found in the supplementary material. We used Bayesian multilevel regression to predict cortical speech envelope tracking (the correlation between predicted brain activity from speech envelope encoding and measured brain activity) from the number of distractors (i.e. condition effect) as well as from individual prediction tendency.

Firstly, we find a negative effect for the number of distractors on envelope encoding in bilateral auditory areas (*b* = −0.009, 94%HDI = [−0.023, −0.002], see Fig. 3A). This indicates that envelope encoding decreases with increasing noise for average prediction tendency. Secondly, we find a positive effect for prediction tendency in right perisylvian areas (namely inferior frontal, precentral, and superior temporal cortex) and left inferior frontal cortex (*b* = 0.011, 94%HDI = [0.002, 0.025], see Fig. 3B). This suggests that individuals with stronger prediction tendency show an increased envelope encoding in these areas. There was also a negative effect for prediction tendency in the left inferior temporal cortex (*b* = −0.008, 94%HDI = [−0.015, −0.001]). Furthermore, our results indicate a positive interaction effect between the number of distracting speakers and prediction tendency in left inferior temporal and inferior frontal cortex (*b* = 0.004, 94%HDI = [0.001, 0.008]). This suggests that as speech gets more difficult to understand the role of prediction tendencies for envelope encoding changes in these left hemispheric areas (see Fig. 3C). However, we also find a negative interaction effect between the number of distracting speakers and prediction tendency in right inferior frontal, mid temporal, and superior temporal areas (*b* = −0.005, 94%HDI = [−0.011, −0.001]). This indicates a right lateralized decreased effect for prediction tendencies on envelope encoding with increasing noise (see Fig. 3C).

To show that the relationship between prediction tendency and the encoding of speech features cannot merely be explained through individual differences in signal-to-noise ratio or differences in task engagement we also calculated “control models.” One model includes the individual decoding accuracy (of sound frequencies in the entropy modulation paradigm) as an additional predictor and the other model includes subjective task engagement ratings. We find that individual decoding accuracy itself cannot predict envelope encoding. Furthermore, the HDI of the beta coefficients for individual decoding accuracy does not overlap with that of prediction tendency (Supplementary Table S2, see online supplementary material). The same applies to subjective task engagement (Supplementary Table S3, see online supplementary material). We therefore conclude that speech tracking effects cannot be explained by differences in pure tone decoding or differences in individual task engagement. In sum, our results suggest an important, albeit differential role of individual prediction tendencies on speech envelope encoding.

### Violations of semantic probability differentially affect speech tracking

**Fig. 4:**
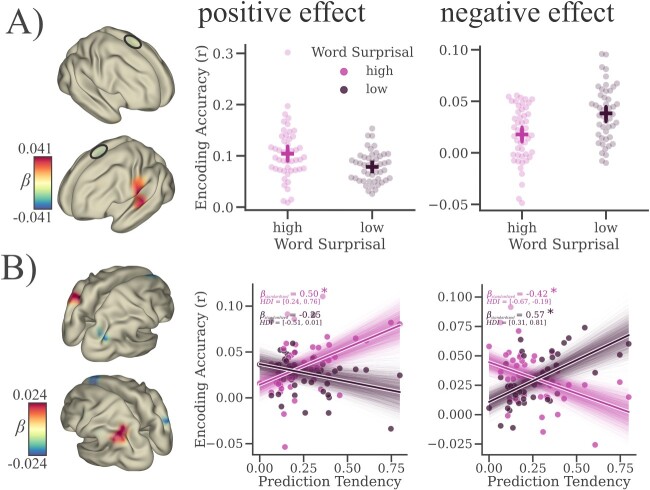
Violations of semantic probability differentially affect speech tracking. A) Lexically identical words differently affect the encoding of the envelope, if they are uttered in an (un-) related context. In the left temporal lobe we notice a positive association between word surprisal and encoding accuracy (the speech envelope was better encoded when words were uttered in an unrelated context). However, there was also a weak negative effect of word surprisal on envelope encoding in right bilateral motor areas (the speech envelope was better encoded when words were uttered in a related context; marked by a black circle). B) Furthermore, there was an interaction effect between individual prediction tendency and word surprisal, suggesting a decreased encoding of semantic violations in the right, vs. an increased encoding of semantic violations in the left hemisphere, that scales with prediction tendency (brain maps show “clusters” significantly different from zero, scatter plots show one-directional model results averaged over all clusters—for details see Methods; shaded areas always indicate 94% HDIs, *N* = 49).

In order to investigate how the beneficial effect of a strong prediction tendency on speech processing in general might be affected by unpredictable events, we also induced rare semantic violations into our paradigm. The following effects on word probability and prediction tendency (as well as all results in Fig. 4) hold true for the clear speech condition only. In a direct comparison of lexically identical words, which have been differentially embedded into the semantic context, we find a positive effect on envelope encoding for words of high surprisal vs. low surprisal in left perisylvian cortex (*b* = 0.024, 94%HDI = [0.005, 0.047], see Fig. 4A). We also find a weak negative cluster showing a decreased encoding of surprising words in bilateral supplementary motor areas (*b* = −0.017, 94%HDI = [−0.031, −0.002], see Fig. 4A). For prediction tendency, again there was an overall positive effect in right superior temporal as well as bilateral inferior frontal cortex for encoding of words of low surprisal (*b* = 0.020, 94%HDI = [0.004, 0.041]). Furthermore, there was a left lateralized positive interaction between word surprisal and prediction tendency, indicating an increase in encoding of surprising words with increased prediction tendency in left angular gyrus (*b* = 0.017, 94%HDI = [0.004, 0.032], see Fig. 4B). Vice versa, we find a right lateralized negative interaction in mid occipital and superior frontal cortex, indicating that, with increased prediction tendency, encoding of surprising words is decreased whereas encoding of unsurprising words seems to be increased in these areas (*b* = −0.018, 94%HDI = [−0.033, −0.003], see Fig. 4B). In addition, our results also show an effect for the number of distracting speakers along with several interaction effects with noise, which are all listed in Supplementary Table S3 (see online supplementary material). In sum, the effects for the number of distractors for words of low surprisal replicate those of the previous section. We also find that effects of word surprisal along with the aforementioned interaction effects seem to diminish with increasing noise. We therefore conclude that the interaction between the influence of word surprisal and individual prediction tendency on encoding is negligible under conditions of high background noise. Instead, we focus our interpretation on the interaction effects of word surprisal and prediction tendency with respect to the clear speech condition.

## Discussion

Based on previous research emphasizing the ubiquity of the predictive brain, the aim of the current study was to investigate the supportive role of strong prediction tendencies in everyday situations such as listening. We propose a link between individual auditory prediction tendency and differences in the processing of spoken language. Across 2 independent paradigms, we connected the individual tendency to preactivate expected sound frequencies to the individual encoding of continuous narrative speech under varying conditions of background noise. Our results demonstrate that speech tracking is related to individual prediction tendency. Furthermore, manipulating contextual predictability of speech by including rare semantic violations allowed us to investigate how this in itself beneficial effect is affected by unpredictable events, showing spatially differentiable interactions.

### Speech tracking is related to individual prediction tendencies

Prediction tendency is often conceptualized as an individual trait, a tendency that varies considerably across individuals and generalizes across situations, and that could be linked to psychiatric disorders such as autism (Pellicano and Burr 2012) and schizophrenia (Corlett et al. 2019). Taking into account its explanatory value for such long-term and highly individualized disorders, predictions have worked their way from feedback connections carrying expected lower-level neural activity (Rao and Ballard 1999) to an individual disposition that forms one of many core dimensions of personality. Here we make no such claim, but we strongly suggest that more emphasis is put on the investigation on the intra-individual stability of prediction tendencies (see Siegelman and Frost 2015 for similar efforts regarding statistical learning). Furthermore, our findings that it (i) seems to generalize across listening situation, and that it is not modulated by (ii) signal-to-noise ratio (decoding accuracy) or (iii) engagement (as shown in our control model in the supplementary material), or (iv) attention (as we are currently investigating by using the same paradigm under different conditions of attention; Demarchi et al. in prep.) favor the general assumption of a “trait” as compared with a “state” dependent measure. So far strong predictive tendencies have been linked to maladaptive auditory phantom perception (e.g. hallucinations and tinnitus, see Corlett et al. 2019 and Sedley et al. 2016). However, the beneficial aspects of prediction tendencies in complex listening situations have received little scientific attention.

Our findings show that the tendency to anticipate low-level acoustic features according to their contextual probability is positively related to the cortical tracking of the speech envelope. Tracking of information conveyed by the speech envelope is critical for speech comprehension and the processing of low-level linguistic features (Vanthornhout et al. 2018; Schmidt et al. 2021). This suggests a potentially supportive role of the tendency to anticipate low-level acoustic features on the processing of naturally spoken language. Expectedly, this effect is most prominent in areas associated with auditory processing and speech perception such as inferior frontal as well as temporal regions of the right hemisphere, but also in Broca’s area of the left cortex (see Fig. 3B). To our knowledge, this is the first study to link speech tracking capabilities to individual differences in prediction tendency. This finding becomes all the more remarkable as our quantification of prediction tendency stems from a very different auditory paradigm that was completely independent of speech processing. Therefore, we can infer some generalizability of individual prediction tendencies across different listening situations. Most importantly, we argue that this link cannot be explained by individual signal-to-noise ratio or other attributes (such as linearity and distributive properties) that are known to have an influence on the performance of classification and encoding algorithms. Firstly, prediction tendency is quantified as the difference in anticipatory predictions between conditions (thus uninformative idiosyncrasies in feature decodability are canceled out) and secondly, we find that individual decoding accuracy (of sound frequencies in the entropy modulation paradigm) itself cannot predict envelope encoding. Furthermore, the HDI of the beta coefficients for individual decoding accuracy does not overlap with that of prediction tendency (Supplementary Table S2, see online supplementary material). Furthermore, by including subjective ratings of task engagement into our control model (Supplementary Table S3, see online supplementary material), we can show that the relationship between individual prediction tendency and speech encoding cannot be explained by individual differences in task engagement.

### The effect of prediction tendency on speech tracking decreases as background noise increases

Regarding how the beneficial effect of prediction tendency on speech envelope tracking is affected by background noise, we find evidence for a dualistic effect showing a clear lateralization. More precisely, we find that the extent to which prediction tendency can explain speech tracking within the right hemisphere decreases with an increasing number of distracting speakers. Thus, showing the strongest effect of prediction tendency on speech tracking while speech is clearly understandable. When simultaneously confronted with multiple speakers at a real cocktail party, the distracting speech streams are often spatially separable from the target stream, but in situations when a spatial segregation is not possible (for example in an online meeting) speech intelligibility is heavily decreased. In our experiment the target and the distractor streams were both presented at the phantom center, creating a similar situation. It is possible that with decreased intelligibility in bottom-up input, predictive processes are impaired from distortions in the auditory feedback-loop. The current results support our assumption that differences in prediction tendency can explain the variability in listening performance under optimal conditions.

However, we also find a left frontal cluster that shows a positive interaction between prediction tendency and background noise. Looking at the slopes in Fig. 3C, this seems to be the result of a decrease in a negative effect of prediction tendency rather than an increase of the positive prediction tendency effect with noise. Thus, the extent to which the left inferior frontal cortex is engaged in speech envelope tracking in individuals with lower vs. stronger prediction tendencies seems to be dependent on background noise (notably this interaction effect only partially overlaps spatially with the reported main effect for prediction tendency). One possible explanation is that differences in prediction tendency may result in differential allocation of attention depending on individual listening effort. For example, Lesenfants and Francart (2020) found that attention plays a crucial role in the cortical encoding of speech and showed an advanced encoding in frontal & fronto-central electrodes with focal attention. So far, differences in challenging listening situations with low signal-to-noise ratio (such as in a cocktail party situation) have often been linked to individual differences in selective attention (Kerlin et al. 2010). Yet, we argue that a predictive theory of individual listening experience is not exclusive but rather complementary to that as predictions are assumed to generate a selective attentional gain to the expected sensory feature (e.g. Marzecová et al. 2018). It seems possible that people allocate attention in a different or more parsimonious way depending on their tendency to rely on predictions.

An alternative (though not mutually exclusive) interpretation is that individuals with weaker auditory prediction tendency have to rely more on the sensorimotor system to integrate acoustic information during speech processing. The general idea is that auditory processing is critically involved in speech production and, vice versa, that motor processes have a modulatory influence on speech perception. An integrative model was proposed by Hickok et al. (2011) in which the motor system promotes auditory forward predictions as part of a sensory-to-motor feedback circuit. This in-built predictive capacity could play a role in supporting listening. Based on this, we speculate that individuals with a stronger tendency to generate purely auditory predictions are less reliant on predictions from the motor system during clear speech perception. This could be reflected in the observed decreased tracking in left Broca’s area for individuals with stronger prediction tendency. We suggest that future research should explore the possibility of an interaction between individual prediction tendency and the sensorimotor system during speech perception in challenging listening situations.

### Violations of semantic probabilities interact with individual prediction tendencies

To test the influence of individual prediction tendencies on changes in semantic probabilities during speech processing, we compared speech envelope encoding between a subset of surprising target words that are semantically unrelated to the preceding context and their lexically identical counterparts for which contextual predictability was not manipulated. Although the benefits of using natural speech with no experimentally induced manipulations have to be acknowledged, one remaining disadvantage is the difficulty in separating top-down from bottom-up influences. In narrative language the acoustic signal is correlated with the higher-level information it conveys and any alteration in speech envelope encoding may be attributed to both aspects (Brodbeck and Simon 2020).

Our results show an increased envelope tracking for surprising words in the left perisylvian cortex. In previous decades, the effects of surprise in speech perception has focused on event-related potentials and the typical N400 component—a negativity at 300–600 ms in central and/or parietal regions. It is well established that unexpected stimuli raise a stronger response (or from another perspective: expected stimuli evoke a reduced neural response), but the relative information content of these responses is still debated (de Lange et al. 2018). Our findings show considerable spatial overlap with previous studies that have focused on surprisal-evoked responses in speech perception (Willems et al. 2016; Frank and Willems 2017), complementing them in 2 ways. Firstly, we provide evidence that these responses are not driven by a rather unspecific error signal but instead actually encode information about the surprising stimulus itself. Our results indicate that the low-level acoustic representation is sharpened for high compared with low surprisal and, importantly, that this sharpening is a consequence of contextual unpredictability and cannot be explained by any differences in bottom-up input. Secondly, even though the impact of predictions on speech perception has widely been emphasized (e.g. Heilbron et al. 2022), the question of how individual prediction tendency interacts with the processing of semantic violations has, to the best of our knowledge, not been investigated so far. Originally, we expected a disrupted encoding of surprising words, scaling with individual prediction tendency. Instead, we find a spatially dissociable interaction effect, indicating the expected interference for surprising words in right occipital and superior frontal regions on the one hand (or hemisphere), but also an increased encoding of surprising words in left angular gyrus with increased prediction tendency on the other.

In the current study, prediction tendency is operationalized by the tendency to preactivate sound frequencies of high probability in a predictable context. Contextual predictability was determined via a first-order markov chain (i.e. each subsequent state depends only on the immediately preceding one). Human language (although see Sainburg et al. 2019 for an example in birdsong) is assumed to follow a more complex, hierarchical non-markovian structure (Chomsky 1956; Sainburg et al. 2019). The current results show that lower-order predictions seem to be relevant in the processing of acoustic speech. Furthermore in continuous speech, predictions happen at a number of different levels such as acoustic, syntactic, semantic etc... Here we focused our analysis on acoustic (i.e. speech envelope) tracking, however, the fact that prediction tendency seems to interact with semantic predictability, independent of acoustics, indicates a potential generalization to higher-order speech processes as well. Future research should focus on the explanatory value of individual prediction tendency for speech processing across all levels along the linguistic hierarchy.

### Limitations

There are several limitations to the interpretation of our results. Firstly, using a regression model, we cannot draw any conclusion on causality. Although the idea that individual prediction tendencies influence cortical speech tracking is certainly one compelling way to explain our results, we cannot rule out other causal agents (such as cognitive abilities) or a reversed directionality. Secondly, we were not able to directly link prediction tendencies to differences in behavioral task performance (due to data loss) or to subjective listening experience. Although one might argue that these variables are of considerable importance for real-life implications, in the current study they are likely to be biased by floor/ceiling effects (see supplementary material). Thirdly, we know that auditory predictions can be coarsely divided into spectral and temporal predictions that are associated to slightly different regions (e.g. Auksztulewicz et al. 2018; Wollman and Morillon 2018) and both play an important, albeit differential, role for language perception and production (Forseth et al. 2020). Here we demonstrate that spectral prediction tendency plays a role in acoustic speech tracking, however, as the sound sequence used to assess that tendency were highly rhythmic (with a fixed rate of 3 Hz), we cannot infer the extent to which this relationship might be driven or modulated by temporal prediction tendency. Finally, we did only focus on one acoustic feature although speech perception encompasses many more linguistic features (such as formants, phonemes, syllables etc.), and even though we demonstrated an effect of word surprisal on low-level encoding, we did not investigate the tracking of such higher-level features directly.

## Conclusion

We support the assumption that predictive processing is an ubiquitous perceptual phenomenon and that it is crucial for continuous speech perception. Furthermore, we argue that the tendency to engage in these predictive processes is different from individual to individual and that this variability can explain differences in listening experience. Importantly, individual prediction tendency can be assessed independently of the speech perception it facilitates, thus we can infer some generalizability of auditory predictions across different listening situations.

## Supplementary Material

supplementary_material_bhac528

## Data Availability

Access to raw data will be made available upon reasonable request.
