# Supplementary material for: Cortical speech tracking is related to individual prediction tendencies
Source: Cereb Cortex. 2023 Jan 9;33(11):6608–19. doi: 10.1093/cercor/bhac528 (PMC10233232; doi:10.1093/cercor/bhac528)
Supplement: supplementary_material_bhac528 [file supplementary_material_bhac528.docx]

# SUPPLEMENTARY MATERIAL

## Stimulus Material

We used audio recordings of excerpts from German books and short-stories (listed in the table below) for our multispeaker listening task. Excerpts were selected prior to recording in such a way that one trial had an approximate reading duration of 3 - 4 min (mean = 3.46 min, range = 3.05 - 4.07 min). In total 27 trials were recorded from the following material:

| Table 1: List of all ebooks and short stories that were used as basis for audio material |
| --- |
|  |
| “Ein zauberhafter Schrebergarten” by Anja Pompowsk^1^ |
| “Winterzauber in der kleinen Keksbäckerei” by Holly Hepburn^1^ |
| “Darius’ Radius” by Maida Thesy^1^ |
| “Die Möwe Jonathan” by Richard Bach^1^ |
| “Die Eskimos: Geschichte und Schicksal der Jäger im hohen Norden by T. Jeier^1^ |
| “Sofies Welt” by Jostein Gaarder^1^ |
| “Die zwei Weiden” and “Der Zaunkönig und die Rose” by Florian C. Pichler^2^ |
| “Der Goldstrauch und 66 weitere Kurzgeschichten für zwischendurch” by Alfred Bekker^2^ |
| “Der Schneesturm” by Nadja Rohner^2^ |
| “Die Burg” by Werner Kistler^2^ |
| “Kishon's schönste Geschichten für Kinder” by Ephraim Kishon^2^ |
| Note: ^1^main stories narrated by a target speaker (3 x ~3min excerpts each), ^2^distractor stories narrated by a distractor speaker (18 x ~3min excerpts in total) |

## Speech tracking effects cannot be explained by differences in pure tone decoding or differences in individual task engagement

To show that the relationship between prediction tendency and the encoding of speech features cannot merely be explained through individual differences in signal-to-noise ratio we added individual (zero-centered) pure-tone decoding accuracy peaks from the entropy modulation paradigm into our model. Envelope encoding was averaged over voxels that showed a significant effect of prediction tendency and used as dependent variable. Model summary statistics (see Table 2) show that individual pure-tone decoding cannot predict envelope encoding results (b = -0.001, 94%HDI = [-0.004, 0.002]) and the posterior probability distribution shows no overlap with the posterior probability distribution of prediction tendency (b = 0.011, 94%HDI = [0.007, 0.014]). We therefore conclude that the influence of individual prediction tendency on speech encoding cannot be attributed to differences in decoding results per se. The same control analysis was calculated including self-reported engagement ratings (median centered) into our model. Model summary statistics (see Table 3) show that individual task engagement cannot predict envelope encoding results (b = 0.001, 94%HDI = [-0.002, 0.003]) and the posterior probability distribution shows no overlap with the posterior probability distribution of prediction tendency (b = 0.012, 94%HDI = [0.009, 0.015]). We therefore conclude that the influence of individual prediction tendency on speech encoding cannot be attributed to differences in task engagement.

| Table 2: Model summary statistics including pure-tone decoding accuracy | | | | |
| --- | --- | --- | --- | --- |
|  | b | sd | hdi 3% | hdi 97% |
| Intercept | 0.047 | 0.001 | 0.044 | 0.05 |
| n distractors | -0.015 | 0.001 | -0.016 | -0.013 |
| prediction tendency | 0.011 | 0.002 | 0.007 | 0.014 |
| n distractors x prediction tendency | -0.005 | 0.001 | -0.006 | -0.003 |
| decoding accuracy | -0.001 | 0.002 | -0.004 | 0.002 |
| n distractors x decoding accuracy | 0.001 | 0.001 | -0.001 | 0.002 |
| prediction tendency x decoding accuracy | 0.001 | 0.001 | -0.002 | 0.003 |
| n distractors x prediction tendency x decoding accuracy | 0.001 | 0.001 | -0.001 | 0.002 |
| Note: Dependent Variable = mean envelope encoding over voxels showing a sign. effect for prediction tendency | | | | |

| Table 3: Model summary statistics including subjective ratings of engagement | | | | |
| --- | --- | --- | --- | --- |
|  | b | sd | hdi 3% | hdi 97% |
| Intercept | 0.047 | 0.002 | 0.044 | 0.05 |
| n distractors | -0.013 | 0.001 | -0.015 | -0.012 |
| prediction tendency | 0.012 | 0.002 | 0.009 | 0.015 |
| n distractors x prediction tendency | -0.004 | 0.001 | -0.006 | -0.003 |
| engagement | 0.001 | 0.001 | -0.002 | 0.003 |
| n distractors x engagement | -0.001 | 0.001 | -0.002 | 0.001 |
| prediction tendency x engagement | -0.002 | 0.001 | -0.005 | 0 |
| n distractors x prediction tendency x engagement | 0.001 | 0.001 | -0.001 | 0.003 |
| Note: Dependent Variable = mean envelope encoding over voxels showing a sign. effect for prediction tendency | | | | |

## Violations of semantic probabilities interact with individual prediction tendencies as well as with background noise level

As we investigated how semantic violations are encoded differently in comparison to their lexical identical counterparts we further included individual prediction tendency as well as the number of distractors into our model. The results can be found in the table below. Note that for most predictors we find a positive as well as a negative effect albeit in spatially different locations.

| Table 4: Model summary statistics for comparison of high vs. low word surprisal | | | | | |
| --- | --- | --- | --- | --- | --- |
|  |  | b | sd | hdi 3% | hdi 97% |
| Intercept | L+R: auditory C. | 0.046 | 0.024 | 0.013 | 0.101 |
| n distractors | L+R: auditory C. | -0.019 | 0.010 | -0.043 | -0.005 |
| prediction tendency | L+R: front.inf./ precentr., R: temp.sup. | 0.020 | 0.010 | 0.004 | 0.041 |
| word surprisal (high > low) | L: postcentr., temp.sup., perisylv.A. | 0.024 | 0.011 | 0.005 | 0.047 |
|  | L+R: SMA | -0.017 | 0.008 | -0.031 | -0.002 |
| n distractors x word surprisal | L: SMA, S.calc., R: temp.mid., occ.mid. | 0.015 | 0.006 | 0.003 | 0.027 |
|  | L: postcentr., R: front.mid. | -0.016 | 0.007 | -0.031 | -0.003 |
| word surprisal x pred. tend. | L: pariet.inf., angular G. | 0.017 | 0.007 | 0.004 | 0.032 |
|  | R: front.sup.med. ,occ.mid. | -0.018 | 0.008 | -0.033 | -0.003 |
| n distractors x pred. tend. | L: occ.sup. | 0.010 | 0.005 | 0.002 | 0.019 |
|  | L: precentr., R: temp.mid./sup., front.mid. | -0.012 | 0.005 | -0.023 | -0.002 |
| n distractors x word surprisal x prediction tendency | L: front.sup.med.,pre-/postcentr., R: occ.mid | 0.014 | 0.006 | 0.003 | 0.027 |
|  | L: perisylv.A., pariet.inf., R: front.inf./mid. | -0.015 | 0.007 | -0.028 | -0.003 |
| Note: Dependent Variable = mean envelope encoding over voxels showing a sign. effect for each predictor | | | | | |

## Behavioural Results

#

**FIGURE 5**

As we were not able to investigate speech comprehension on a behavioural level we had to use self reported ratings on difficulty (on a 5-point likert scale) as a proxy for individual perception. We clearly find an effect for the number of distractors indicating that the task was perceived more difficult with increasing background noise (b = 1.251, 94%HDI = [1.173, 1.325]). There was no visible effect for individual prediction tendency (see **Fig. 5A** and Table 4) indicating that across subjects the task was perceived equally difficult across subjects with different prediction tendencies. It should be noted, however, that the ratings for the dist-0 condition and the dist-2 condition show a pronounced floor and ceiling effect respectively (see **Fig. 5B)**. Further we investigated whether background noise and/or individual prediction tendency had an effect on task engagement. We found no difference in self reported engagement between conditions (b = -0.059, 94%HDI = [-0.124, 0.005]) and no effect for individual prediction tendency on engagement (b = -0.096, 94%HDI = [-0.318, 0.116]; see **Fig. 5B** and Table 5).

| Table 5: Model summary statistics for perceived task difficulty | | | | |
| --- | --- | --- | --- | --- |
|  | b | sd | hdi 3% | hdi 97% |
| Intercept | 1.941 | 0.083 | 1.780 | 2.094 |
| n distractors | 1.251 | 0.041 | 1.173 | 1.325 |
| prediction tendency | 0.090 | 0.082 | -0.061 | 0.244 |
| n distractors x prediction tendency | -0.043 | 0.040 | -0.117 | 0.034 |
| Note: Dependent Variable = mean rating of task difficulty over blocks (on a 5-point likert scale) | | | | |

| Table 6: Model summary statistics for self reported engagement | | | | |
| --- | --- | --- | --- | --- |
|  | b | sd | hdi 3% | hdi 97% |
| Intercept | 3.889 | 0.120 | 3.659 | 4.108 |
| n distractors | -0.059 | 0.035 | -0.124 | 0.005 |
| prediction tendency | -0.096 | 0.118 | -0.318 | 0.116 |
| n distractors x prediction tendency | -0.030 | 0.035 | -0.097 | 0.034 |
| Note: Dependent Variable = mean rating of engagement over blocks (on a 5-point likert scale) | | | | |

#

**FIGURE CAPTIONS**

***Fig. 5:*** *Background is affecting subjective ratings of difficulty in a multi- speaker listening task.* ***A)*** *Perceived task difficulty (indicated by subjective ratings on a 5-point likert scale) increases with the number of distractors and is not affected by individual prediction tendency.* ***B)*** *Engagement (indicated by subjective ratings on a 5-point likert scale) is neither affected by the number of distractors nor by individual prediction tendency.*
